# Professional Life During COVID-19 Crisis: An Emotional and Ethical Dilemma for the Medical Staff

**DOI:** 10.1017/dmp.2020.394

**Published:** 2020-10-22

**Authors:** Maryam Chehrehgosha, Zahra Royani

**Affiliations:** 1 Candidate in Gerontology, Department of Geriatrics, University of Social Welfare and Rehabilitation Sciences, Tehran, Iran; 2 Laboratory Sciences Research Center, Golestan University of Medical Sciences, Gorgan, Iran; 3 Laboratory Sciences Research Center, Faculty Member of Department of Surgical Technology, Paramedicine School, Golestan University of Medical Sciences, Gorgan, Iran

**Keywords:** crisis, COVID-19, ethical dilemma, medical staff, professional life

At the end of 2019, China reported an unknown outbreak of pneumonia to the World Health Organization (WHO), and on March 11, 2020, the WHO declared the coronavirus disease (COVID-19) a pandemic.

Hospital personnel are constantly at the forefront of epidemics, risking their lives to perform their duties and work in stressful environments while focusing on helping the patients. Medical staff provide safe care and services, knowing that they are at high risk for infection^1^; however, their rights as citizens, like those of others in society, might be neglected in the midst of such dilemmas and challenges because, in a crisis, we see them as the tireless medical staff.

During the COVID-19 pandemic, the health professionals have to work in isolation wards in hospitals, and they explain how working in the isolation wards for a long duration “created a sense of collective hysteria and made sense of disappointment in medical staff.”^2^

Moreover, lack of knowledge about COVID-19, unbearable workload, and insufficient availability of personal protective equipment (PPE) may lead to the different levels of psychological pressure among the medical staff.^3^

Due to limited resources, detaching a patient from a ventilator to save another leads to an ethical dilemma in the medical staff. For example, the elderly or frail patients infected with COVID-19 need more intensive care for a longer duration while younger patients have a better chance of recovery using on time treatment.^4^

The body and soul of the medical staff were tired during this crisis, but they have shown enormous compassion. In Wuhan (China), a nurse heard about her mother’s death and returned to patient care nonetheless, and some nurses shaved their heads to prevent cross-infection and to facilitate doffing and donning all PPE. In Italy, a medical student passed his final exam and went to the heart of the outbreak in Bergamo. In the UK, a 68-year-old retired physician returned to London to help fight the pandemic.^5^

We called them life-saving heroes in appreciation for the health care, but we forget that their selfless acts might have some negative consequences. Using the term *hero* implies that they are invincible and, despite all the hardships, they can continue to provide care and services with power. However, we omit the concept that they are human, that they have a threshold of physiological and psychological capacity, and, like other people, may be frustrated, exhausted, and also feeling fear.

## CONCLUSION

Although the COVID-19 pandemic will end one day and treatment will be discovered, another crisis that has gone unnoticed is the exhausted souls and tired bodies of the medical staff, which should be tended to after the days of the COVID-19 pandemic. The psychological trauma of the medical staff may be forgotten when the pandemic is well under control. After this critical period, special attention should be paid to providing mental health care for the medical staff.
